# The gap between self-reported and objective measures of disease status in India

**DOI:** 10.1371/journal.pone.0202786

**Published:** 2018-08-27

**Authors:** Ilke Onur, Malathi Velamuri

**Affiliations:** 1 School of Commerce, University of South Australia, Adelaide, Australia; 2 Chennai Mathematical Institute, Chennai, India; University of Texas Medical Branch at Galveston, UNITED STATES

## Abstract

Researchers interested in the effect of health on various life outcomes (such as employment, earnings and life satisfaction) often use self-reported health and disease status as an indicator of true, underlying health status. Self-reports appear to be reasonable measures of overall health. For example, self-assessed overall health has been found to be a reliable predictor of mortality. However, the validity of self-reports is questionable when investigating specific diseases such as diabetes and hypertension. A small and nascent body of research comparing self-reported status on certain diseases with the true status based on clinical diagnoses has found significant gaps. These validation exercises predominantly use data from high-income countries. In this paper, we use survey data from India to compare self-reports of disease prevalence to diagnostic tests conducted on the same individuals. We focus on hypertension and lung disease, two of the primary causes of death in India. We find that self-reported measures substantially understate the true disease burden for both conditions. The attenuation bias from using self-reports is over 80 percent for both diseases, and bigger than estimates from high-income countries. We test and reject the hypothesis that self-reports of the disease status are identical to the true disease status in expectation. We identify characteristics associated with false negative reporting (reporting not having the disease but testing positive for it) for both diseases. The large awareness gap between self-reports and true disease burden indicates multiple deficiencies in India’s public health policy. The survey data depicts limited access to medical facilities, high levels of health illiteracy, low rates of health insurance, and other barriers related to poverty and lack of equity in the delivery of health services. These factors prevent timely intervention for managing health and controlling disease, invariably leading to morbidity and often to premature death.

## Introduction

A large body of research across disciplines documents the influence of health status on a number of economic and social outcomes such as income, employment status, job satisfaction, marital status and life satisfaction [1]. Much of this research relies on self-reports to measure health status. Evidence from both developed and developing countries indicates that self-assessed general health is a reliable predictor of mortality and of certain diseases such as cardiovascular disease [2–5]. But some other studies have documented sizable gaps in the prevalence of other diseases, as measured by self-reported versus true (clinically diagnosed) disease status [6, 7], with the magnitude of the gap varying by disease. This nascent literature is predominantly based on data from high-income countries.

India accounts for about 20 percent of the global burden of diseases. However, the country’s share of hospital beds and doctors is only 6 and 8 percent respectively [8]. This situation is exacerbated by spatial inequality of access; about 72 percent of the population lives in rural areas while the majority of healthcare facilities is based in urban areas [9]. The public health system has not stepped up to meet these challenges [10]. All these factors contribute to large spatial inequalities in health literacy and uneven progress in improving the health status of the population [11, 12].

In this paper, we compare prevalence rates of two disease conditions—hypertension and lung disease—as measured by self-reports and by reports from diagnostic tests administered to the same individuals, using a dataset on older (45+) individuals in India. Our objectives are: (1) to document the nature and extent of errors in self-reports for the two conditions; (2) to quantify the bias arising from using self-reports instead of objective measures in statistical analyses; and (3) to analyze the determinants of false negative reporting (reporting not having the disease/condition but testing positive for it), taking account of the censored nature of the dependent variable.

### Related literature

Evidence from high-income countries suggest that the magnitude of attenuation bias from using self-reported health measures is large. Baker et al. documented errors in self-reported status for 13 disease categories, by linking data from a Canadian household survey to health administrative data in the province of Ontario [7]. In all categories, they recorded a false negative rate of over 50 percent. In comparison, rates of false positives were smaller, but relatively larger for conditions that individuals tend to self-diagnose, such as migraine.

Johnston et al. used the health survey for England to examine the gap between self-reported and clinically tested hypertension [6]. They found 28% under-reporting on average, and estimated an attenuation bias of 68 percent. Their estimates indicated that while household income had no effect on self-reports, a 1 log-point increase in household income lowered clinical hypertension by about 1%. They also found that the probability of false negative reporting declines with income.

Suziedelyte and Johar examined the consistency of self-reports on four invasive surgical procedures over the previous 5 years [13]. The procedures were knee replacement, removal of gall bladder, removal of prostrate and hysterectomy. They linked self-reports from survey data in the Australian state of New South Wales with hospital records on all admissions in the state. They also compared self-reported hypertension with claims data for prescription drugs for treating hypertension, and hypertension diagnoses in hospital admission records in the 12 months after the survey date. Misreporting was common in their data, with higher rates of over-reporting (false positives) for all procedures except prostrate surgery, ranging from 26% to 29%. For hypertension, over-reporting accounted for about 32% while the rate of under-reporting was about 28%. Respondent characteristics could explain little of the variation in reporting error.

Evidence on the degree of misreporting of health conditions in developing countries is meagre due to data limitations. An exception is [14] who compared self-reported rates of five common non-communicable diseases (NCDs) in India from the Study on Global Ageing and Adult Health, with age-adjusted prevalence rates of these diseases. They estimated disparities based on household wealth and education categories. They found evidence of significant under-reporting among low socioeconomic groups. They infer that this is due to poor access to high quality healthcare in India.

Cramm et al. used the same data source as this paper to examine the association between self-reported health and objective health based on tests conducted by the survey staff [15]. However, they did not compare subjective and objective indicators for the same disease. Their subjective health measures were self-rated health and dependence in activities of daily living, while their objective measures were test-diagnosed results for lung disease and grip strength. They found a weak correlation of at most 14%, between subjective and objective health indicators. They also found that respondents tend to overstate their health status, relative to what the objective measures indicate.

In contrast to [15], we compare subjective and diagnostic reports of two disease conditions—hypertension and lung disease—for the *same* individuals. This provides a validation exercise for the self-reported data. Our paper also differs from [14] in that we present estimates of bias that arise from using self-reports in place of objective measures. We examine characteristics associated with false negative reporting for both diseases.

### Hypertension and lung disease in India

The two primary causes of death in India are coronary heart disease (CHD) and lung disease. A key risk factor for CHD is hypertension. In India, hypertension leads the list of NCD risks, estimated as a factor in about 10 percent of all deaths [16]. Moreover, prevalence rates among adults have risen dramatically over the past three decades and are expected to rise further [16–18].

Hypertension can often be asymptomatic, even at elevated levels of blood pressure [6]. Thus, though it is inexpensive and easy to detect, it is likely to remain undiagnosed if the individual is not accustomed to visiting a doctor periodically. This leads to preventable morbidity and deaths.

Lung disease is the second biggest cause of deaths in India. Lung disease refers to many conditions affecting the lungs, including asthma, chronic obstructive pulmonary disease (COPD), infections like pneumonia, influenza and tuberculosis, and lung cancer. The age-adjusted death rate due to lung disease, at 127 per 100,000 of population, places India at #1 in the world by this criterion [19]. While many conditions comprising lung disease such as respiratory illness come under NCDs, tuberculosis (TB) is an infectious disease. India also ranks first among the list of 22 countries constituting the highest TB burden in the world, and accounts for one-fifth of the global incidence of TB [19].

Lung disease is often associated with some typical symptoms such as cough, breathlessness and fever. However, these symptoms can be confused with those of milder conditions such as influenza, and ignored. A visit to a specialist is required to diagnose lung disease. Problems of access to specialist medical care and financial considerations prevent the prompt diagnosis of this very serious disease.

## Materials and methods

We use the 2010 pilot wave of the Longitudinal Aging Study of India (LASI) survey. This survey is designed as a sister survey to the Health and Retirement Survey (HRS) of the United States and similar surveys in Europe, Asia and South America. LASI is designed to be a nationally representative longitudinal survey of India’s aging population 45 years or older and their spouses. LASI will follow 30,000 individuals over time, surveying them once every 2 years. The survey covers demographic, health, economic, and psychosocial topics relevant to studies on aging. Data from the first wave is yet to be released for public use.

The pilot wave of LASI was conducted in 2010 in four states—Punjab and Rajasthan in the North and Kerala and Karnataka in the South. The survey used the 2001 Indian census to draw a representative sample from the four selected states, and in each state, two districts were selected at random. In each of these districts, eight primary sampling units (PSUs) were chosen. In small rural PSUs (with fewer than 500 households), a two-stage sampling procedure was used while larger rural and urban PSUs used a three-stage procedure. This sampling procedure created a sample size of 950 households. Weights were created using the inverse probability of selection combined with household and individual response rates [20]. This complex survey design introduces more sample-to-sample variability than simple random sampling. In our descriptive statistics as well as regression analyses, we adjust standard errors to reflect the larger survey error.

The LASI questionnaire comprises three main components: the household interview, the individual interview and a biomarker module [21]. The household module surveys socio-demographic characteristics of the household, household finances, expenditure, consumption, and assets. This module can be answered by any knowledgeable household member 18 years of age or older. For the individual interview, only those who were at least 45 years of age at the time of the survey were eligible. The individual questionnaire was completed by any consenting, age-eligible person in the household and his/her spouse. The age criterion did not apply to the spouse. In total, 1,683 individuals completed the individual module. The purpose of the biomarker module is to provide objective health measures. This module includes anthropometric measures, blood pressure readings, vision, lung function and physical functioning tests, as well as some biomarkers based on the analysis of dried blood spots (DBS) collected from the respondents. Of the 1,683 respondents, only 1,311 completed the biomarker module. We restricted the analysis in this paper to respondents who were between 45-85 years of age at the time of interview. This reduced the sample size to 1,149.

Risk factors for many chronic and communicable diseases are associated with poor socioeconomic conditions [12, 22]. We thus include a number of variables in our analysis that proxy for socioeconomic status. Caste and religious identities are particularly salient in India. The Scheduled Castes (SCs) and Scheduled Tribes (STs) are two historically disadvantaged groups recognized in the Constitution of India. Other Backward Class (OBC) is a collective term used by the Government of India to classify groups, other than SCs and STs, which are also educationally, economically and socially disadvantaged. The residual category (None/Other) includes the ‘forward’ castes that enjoy high socioeconomic status. Hindus are the dominant religious group in India while Muslims, Christians and Sikhs are the prominent religious minorities. Our set of chosen individual characteristics comprise marital status, number of children, educational attainment, work status, health indicators, health insurance status and lifestyle variables. In addition to per capita expenditure, we include certain household characteristics that reflect socioeconomic status such as indicators for whether the household has electricity, uses good quality fuel for cooking, has indoor plumbing and a toilet inside the compound.

The health module of the individual questionnaire provides anthropometric information. This module also asks the respondent whether s/he has been diagnosed with an extensive list of diseases. We focus on 2 specific conditions: hypertension and lung disease. We chose these conditions because we can compare the responses to objective measures of the corresponding condition from the biomarker module. Questions pertaining to these conditions were phrased as follows: (1) “Has any health professional ever told you that you have high blood pressure or hypertension?”; (2) “Has any health professional ever told you that you have chronic lung disease such as chronic bronchitis or emphysema?”. If a respondent answered in the affirmative for any of these questions, survey staff asked some follow-up questions such as what type of doctor (allopathic, homeopathic etc.) made the diagnosis and whether respondent is currently on medication/treatment for the condition.

The questions on self-reports are retrospective and rely on the respondents’ memory for a correct response. Since the survey covers older individuals, the accuracy of responses may be of particular concern. We can partially address this concern by controlling for objective test results on episodic memory that are reported for each respondent. Episodic memory refers to the ability to consciously recall specific events and situations from the past, and is considered one of the major cognitive capacities enabled by the brain. Episodic memory loss is usually the first symptom of Alzheimer’s disease. The LASI survey staff read aloud ten words to respondents and asked them to recall these words when the interviewer finished (immediate recall). Respondents were given a score between 0 and 10, depending on how many words they recalled. They were again asked to recall these words at the conclusion of the cognitive functioning tests (delayed recall), with the same scoring pattern. We added the scores for immediate and delayed recall, to create a summary score for episodic memory, ranging from 0 to 20. We use the standardised measure of this score as our measure of memory function.

For the biomarker module, three readings of blood pressure were taken for each respondent. For our main analysis, we averaged the three systolic and three diastolic readings, and coded the respondent as suffering from high blood pressure (BP) or hypertension if the average systolic was over 140 or the average diastolic was over 90. We tested the robustness of our empirical results by using different thresholds/measures for categorising hypertension. There is some support in the medical literature for using higher thresholds for individuals who do not present any other risk of cardiovascular disease [23]. Our second definition used an average systolic reading of over 160 or an average diastolic of over 100. For our third definition, we used the same thresholds as the first definition (140/90) but dropped the first blood pressure reading and used the average of the second and third readings only. This is a common practice in the medical literature that allows for the possibility that the first reading might be higher than normal either because the respondent is nervous or because of physical exertion just before the interview began [6].

Trained survey staff used a spirometer to conduct lung function tests. The forced expiratory volume in one second (FEV1) measures the volume of air (in litres) forcibly exhaled in the first second, while the forced vital capacity (FVC) is the maximum volume of air (in litres) forcibly exhaled out of the lungs until no more can be exhaled. The FEV1/FVC ratio (or FEV1%) is a useful indicator of airflow obstruction. Three readings are available for the FEV1/FVC ratio, which we average over to obtain our measure of FEV%. We follow the criterion used by the global initiative for chronic obstructive lung disease (GOLD) of FEV1% < 70 as an indicator of chronic obstructive lung disease (COPD), regardless of age. While 78% of the sample completed the lung function test, some of the test results were out of range due to recording errors. Results for these records are coded as missing in the survey data. Thus we have valid lung function test results for only about 50% (842 respondents) of the overall sample. When we impose sample restrictions, this number is further reduced.

The cardiovascular disease (CVD) risk, pulse rate and Epstein-Barr virus (EBV) antibody levels are variables that we use as exclusion restrictions in our empirical analysis of false negative reporting. We describe these in more detail in the section titled ‘False negative reporting’ below.

### Measurement error in self-reported health and associated bias

We attempt to quantify the ‘measurement error’ in the self-reported hypertension and lung disease status. We assume that the disease status as reported in the biomarker module is the ‘truth’ and we measure the error as the difference between the self-reported status (*S*) and the true status (*T*). The analysis is based on [24] and [7].

Suppose we want to estimate the following model:

$$\begin{array}{c} y=X\beta +\epsilon , \end{array}$$ (1)

However, one of the variables in the *X* vector, *T*, is not observed. Instead, we only observe *S* where *S*_*i*_ = *T*_*i*_ + *u*. That is, *T* is measured with error, but we assume that *cov*(*ϵ*, *u*) = 0. In the case of classical measurement error (CME), *u* is also uncorrelated with *T*. Since both *S* and *T* are dichotomous variables, the measurement error is not CME in our case. If *T* = 1, then *u* ≤ 0, and if *T* = 0, then *u* ≥ 0. This means that the errors are mean-reverting, implying that *cov*(*T*, *u*) < 0. For this case, the proportional bias in estimating *β* is equal to the regression coefficient from a hypothetical regression of *u* = *S* − *T* on *X*. When *S* is the only explanatory variable ([*X*] = [*S*]), this is equivalent to $b_{uS}=\frac{cov(u,S_{i})}{var(S_{i})}$. We report *b*_*uS*_ for both diseases.

We next follow [7] for testing the hypothesis that self-reports of disease status are unbiased estimators of the true disease status. The tests are based on ordinary least squares (OLS) and bivariate probit regressions. We estimate OLS regressions for hypertension and lung disease with the error (*S* − *T*) as our dependent variable, a vector of control variables and a constant. The control variables comprise individual and household characteristics that reflect risk factors for the disease, including socioeconomic characteristics of the household. The test statistic is an F-test for the joint significance of the control variables. In the bivariate probit regressions, our dependent variable is *S* for one equation and *T* for the other, with the same set of control variables used for the OLS regressions. We test the hypothesis that *E*[*S* − *T*|*X*] = 0, based on an F-test that the estimated parameters on *X* in the two equations are equal.

The medical literature identifies age and lifestyle factors as risk factors for hypertension [25]. Lifestyle factors include excessive consumption of dietary salt, smoking, alcohol consumption, high BMI, physical inactivity and a diet deficient in fruits and vegetables. There is evidence suggesting that education [26, 27] and household income [6] lower the propensity for hypertension. The LASI does not contain information on dietary habits of respondents. But we are able to control for the other risk factors and moderating characteristics.

Risk factors for chronic lung disease include smoking, indoor air pollution caused by biomass fuel used for cooking, outdoor air pollution, occupational exposure to dust/gas, respiratory-tract infections during childhood and chronic asthma [28]. Many of these factors are in turn correlated with poor socioeconomic status. While we do not have information on childhood disease history or occupational exposure to pollutants, we do control for type of fuel used for cooking, urban living (that is likely to be correlated with outdoor air pollution), smoking behavior and socioeconomic variables in estimating reporting error regressions for lung disease. Following [20], we define cooking fuel to be of good quality if the household uses any of the following fuels for cooking: coal, charcoal, natural gas, petroleum, kerosene, or electric. In our statistical analysis, we use this definition of good cooking fuel. However, guidelines issued by the World Health Organization (WHO) consider coal and charcoal to be highly polluting and do not include these among recommended fuels for cooking [29]. We therefore use an alternative definition of good quality cooking fuel that excludes coal and charcoal. We test all our results for robustness to the use of this alternate definition.

### False negative reporting

We also estimate models of false negative reporting (*S* = 0|*T* = 1), to identify characteristics that might be associated with such errors. There is a methodological issue with modeling false negative reporting, which arises from the fact that we only observe such errors for individuals diagnosed with the corresponding disease (*T* = 1). For those without the disease (*T* = 0), we do not observe what they would report in the counterfactual scenario. We follow the approach outlined in Johnston et al., of using a censored probit regression model to address this sample selection issue [6]. Van de Ven and Van Praag describe the censored method for addressing sample selection in detail [30]. We outline the model below.

The censored probit model comprises two latent variables: $y_{1}^{j*}$, an individual’s likelihood of having the disease *j*, {j = hypertension, lung disease}, defined as follows:

$$\begin{array}{c} y_{i1}^{j*}=z_{i1}^{j^{\prime }}\alpha_{1}^{j}+\epsilon_{i1}^{j}, \end{array}$$

and $y_{2}^{j*}$ measuring the likelihood of false-negative reporting:

$$\begin{array}{c} y_{i2}^{j*}=z_{i2}^{j^{\prime }}\alpha_{2}^{j}+\epsilon_{i2}^{j}. \end{array}$$

We then have $y_{i1}^{j}=1$ if $y_{i1}^{j*}>0$, and $y_{i2}^{j}=1$ if $y_{i1}^{j*}>0$ *and* $y_{i2}^{j*}>0.$

Thus, for hypertension, *y*_*i*1_ equals 1 if individual *i* has hypertension and zero otherwise, and *y*_*i*2_ equals 1 if *y*_*i*1_ = 1 and *S*_*i*_ = 0, and zero otherwise. The vectors *z*_1_ and *z*_2_ comprise observed socio-demographic characteristics of the individuals in the sample, while *ϵ*_1_ and *ϵ*_2_ are distributed bivariate standard normal with covariance *ρ*: $\left(\epsilon_{1}^{j},\epsilon_{2}^{j}\right)∼N_{2}\left(0,0,1,1,\rho \right)$. The covariance parameter *ρ* captures the impact of unobserved characteristics that might affect both the propensity of having the disease and the propensity to report not having it. We need valid exclusion restrictions to identify the model. The LASI data has no information to indicate whether individuals have a genetic pre-disposition to either disease. Instead, we use other variables that could potentially serve as exclusion restrictions.

The C-reactive protein (CRP) concentration in blood and the pulse reading (heart rate) are two independent predictors of cardiovascular disease (CVD). CRP is a stable and sensitive biomarker for systemic inflammation; an elevated level of CRP in the blood, above the medically-accepted threshold of 3 mg/L, is indicative of infections in the past and is considered an independent risk factor for CVD [31–33]. Pulse/heart rate is yet another independent risk factor for CVD [34]. Since both these variables are risk factors for CVD, they are likely to be correlated with hypertension. At the same time, there is no reason to expect these variables to influence the self-reports, one way or the other. Hence we use these two variables as instrumental variables (IVs) for hypertension.

Exposure to passive smoking and measured Epstein-Barr virus (EBV) antibody activity are our chosen IVs for lung disease. The LASI asks each respondent, “Does any usual member in your household smoke usually inside the home?”, with a valid response being a Yes/No. Those exposed to passive smoking are likely to be at greater risk of lung disease. The Epstein-Barr Virus (EBV) antibody titer is a reliable measure of cellular immune function. EBV is a member of the herpes virus family. Over 90% of the world’s population carry this virus, however, it doesn’t affect most individuals over their lifetimes. For the EBV to remain in a latent state, a well-functioning immune system is crucial [35]. While there are no clinically accepted thresholds for this measure, elevated levels of EBV antibodies indicate a compromised immune system, making individuals vulnerable to many diseases including certain types of lung diseases [36, 37].

## Results

Table 1 presents the socio-demographic characteristics of the overall sample, as well as the samples comprising those with test-diagnosed hypertension and lung disease status as measured by our criteria. The summary statistics highlight some differences in the characteristics of the full sample versus the disease samples, and between the two disease samples. For example, women are slightly over-represented in the overall and hypertension samples, but are about 18 percentage points less likely to be in the lung disease sample. The incidence of both diseases is higher in the urban areas; around 28% of those diagnosed with either disease live in urban areas, relative to the 25% share of the full sample. Being married appears to lower the risk of hypertension by about 8 percentage points but does not seem to make a difference for those with lung disease. There are some significant differences across religious groups in the prevalence of hypertension but caste does not appear to be associated with the prevalence of either disease.

**Table 1:** Characteristics of the sample.

Variable	Full sample	Hypertension (H)			Lung disease (L)		
		H = 1	H = 0	Difference	L = 1	L = 0	Difference
**Demographic characteristics**							
Age structure (%)							
Age 45-54	47.3	39.2	53.1	-0.14***[0.04]	53.8	53.2	0.01 [0.05]
Age 55-64	27.2	28.6	26.6	0.02 [0.03]	24.1	28.3	-0.04 [0.04]
Age 65-74	17.9	22.1	14.9	0.07**[0.03]	16.0	13.3	0.03 [0.03]
Age 75-85	7.5	10.1	5.4	0.05**[0.02]	6.0	5.2	0.01 [0.02]
Females (%)	51.9	52.9	51.3	0.02 [0.04]	35.8	53.6	-0.18*** [0.04]
**Socio-economic characteristics**							
Urban residence (%)	25.0	27.9	23.2	0.05 [0.04]	28.6	28.3	0.0 [0.05]
Caste (%)							
SC	13.7	11.7	15.5	-0.03 [0.03]	11.8	13.6	-0.02 [0.02]
ST	14.8	16.3	13.1	0.03 [0.03]	13.0	10.8	0.02 [0.05]
OBC	38.6	38.5	38.7	-0.0 [0.03]	42.2	41.5	0.01 [0.05]
None/Other	33.0	33.4	32.7	0.0 [0.03]	33.0	34.1	-0.01 [0.04]
Religion (%)							
Hindu	76.3	73.6	78.1	-0.05*[0.03]	82	77	0.05 [0.04]
Muslim	7.3	9.1	5.9	0.03**[0.01]	6.1	8.0	-0.02 [0.02]
Christian	6.8	5.7	7.7	-0.02 [0.02]	7.2	8.5	-0.01 [0.02]
Sikh	8.2	9.9	7.1	0.03**[0.01]	4.2	5.8	-0.02 [0.02]
Other	1.4	1.7	1.2	0.01 [0.01]	1.0	1.0	-0.0 [0.01]
Currently married (%)	79.3	74.4	82.3	-0.08**[0.03]	83.3	83.3	0.0 [0.04]
# children	3.14 (1.96)	3.19 (2.1)	3.08 (1.84)	0.11 [0.17]	3.07 (1.72)	3.05 (1.75)	0.02 [0.15]
Any children dead	14.2	17.0	12.4	0.05*[0.03]	14.0	13.5	0.01 [0.03]
Fully literate (%)	49.8	50.6	49.6	0.01 [0.04]	58.7	61.6	-0.03 [0.05]
Schooling (Years)	4.33 (4.9)	4.21 (4.66)	4.47 (5.1)	-0.27 [0.43]	5.61 (5.31)	5.31 (4.92)	0.3 [0.51]
Education (%)							
No education	47.3	45.5	48.2	-0.03 [0.04]	36.9	35.9	0.01 [0.04]
< 6 years	14.9	18.1	12.4	0.06**[0.03]	15.5	15.8	-0.0 [0.03]
6–12 years	33.2	33.5	33.4	0.0 [0.03]	39.0	42.8	-0.04 [0.05]
> 12 years	4.6	2.9	6.0	-0.03 [0.03]	8.6	5.5	0.03 [0.03]
Ever worked (%)	32.4	31.9	33.7	-0.02 [0.03]	41.5	36.5	0.05 [0.04]
Log expenditure pc	10.36 (2.28)	10.3 (2.3)	10.41 (2.23)	-0.11 [0.21]	10.48 (2.24)	10.43 (1.98)	0.05 [0.2]
No health insurance (HI)	92.2	92.1	92.0	0.0 [0.02]	86.4	95.6	-0.09***[0.03]
Don’t know what HI is	48.7	49.5	48.3	0.01 [0.04]	45.9	51.2	-0.05 [0.05]
Good cooking fuel^@^	41.8	45.0	40.6	0.04 [0.04]	39.5	45.0	-0.06 [0.04]
Good cooking fuel-1^@@^	38.4	42.8	35.9	0.07* [0.04]	36.2	40.5	-0.04 [0.04]
Indoor plumbing	37.5	41.7	35.4	0.06 [0.05]	36.0	38.4	-0.02 [0.05]
Electricity	84.4	85.1	85.1	-0.0 [0.04]	86.1	86.5	-0.0 [0.05]
Toilet inside house	65.8	67.1	65.6	0.02 [0.04]	69.6	73.7	-0.04 [0.05]
**Lifestyle variables**							
Ever smoked	21.4	20.4	22.1	-0.02 [0.03]	29.8	19.9	0.1**[0.04]
Current smoker	17.2	17.1	17.1	-0.0 [0.03]	23.7	16.3	0.07**[0.04]
Former smoker	4.4	3.2	5.0	-0.02 [0.1]	6.1	3.6	0.03 [0.02]
Ever drank	13.8	13.0	14.4	-0.01 [0.02]	21.1	11.8	0.09***[0.03]
Current drinker	9.1	8.6	9.6	-0.01 [0.02]	12.4	8.3	0.04 [0.03]
Former drinker	4.5	4.4	4.7	-0.0 [0.01]	8.7	3.4	0.05***[0.02]
Any exercise	34.5	33.3	36	-0.03 [0.02]	38.6	39.5	-0.01 [0.05]
Heavy exercise	22.7	21.9	23.9	-0.02 [0.02]	24.0	30.4	-0.06*[0.03]
Moderate exercise	11.6	11.4	12.0	-0.01 [0.02]	14.6	9.0	0.06*[0.03]
Passive smoking	27.2	26.9	27.2	-0.0 [0.03]	30.2	24.9	0.05 [0.04]
**Objective health measures**							
Overweight^#^	16.6	18	15.7	0.02 [0.03]	11.5	19.9	-0.08**[0.03]
Obese^##^	6.4	7.9	5.3	0.03*[0.01]	4.9	5.7	-0.01 [0.02]
CVD risk^+^	31.5	35.3	28.5	0.07*[0.04]	28.1	29.3	-0.01 [0.04]
Pulse/heart rate	80.3 (13.5)	83.4 (14.5)	78.0 (12.3)	5.37***[0.97]	78.9 (14.5)	78.9 (13.5)	-0.01 [1.5]
EBV levels^++^	113.0 (62.8)	113.1 (63.6)	113.9 (62.8)	-0.8 [4.27]	106.1 (58.9)	118.1 (64.8)	-12.0***[3.74]
Episodic Memory (0-20)	8.69 (3.48)	8.72 (3.58)	8.65 (3.42)	0.07 [0.26]	9.08 (3.49)	9.02 (3.42)	0.06 [0.36]
Observations (N)	1,149	483	638		242	335	

*Note:* Standard deviation in () parentheses, standard error in [] parentheses;

^@^—Household uses either coal, charcoal, natural gas, LPG, kerosene or electricity for cooking;

^@@^—Household uses either natural gas, LPG, kerosene or electricity for cooking;

^#^ BMI in [25–30] range;

^##^ BMI >30;

^+^C-reactive protein concentration in blood <3 mg/L;

^++^Epstein-Barr virus antibody levels.

*—significant at the 90% level;

**—significant at the 95% level;

***—significant at the 99% level

Less than half the sample is fully literate, though the rate is higher among those with lung disease. The latter group also reveals relatively higher educational levels. The average household per capita expenditure is about 19 percent (0.18 log points) higher among those with lung disease, relative to the sample of hypertensives as well as to the overall sample. Interestingly, they represent a lower share of households using good cooking fuel and having indoor plumbing, relative to the overall average and to the hypertension sample.

A little over 20 percent of the full sample has ever smoked, about 14 percent has ever had an alcoholic drink and about 35 percent do some physical exercise. As expected, smoking as well as exposure to smoking is more prevalent among those with lung disease. The share of current smokers is about 7 percentage points higher among those with lung disease relative to those without. Among hypertensives, the share of obesity is about 3 percentage points higher, and the risk of cardiovascular disease (CVD) is about 7 percentage points higher compared to those without hypertension.

In general, the high rates of illiteracy and low levels of educational attainment indicate that health illiteracy may be a major constraint for achieving a healthy and disease-free status for large sections of the Indian population. The fact that 92% of the sample has no health insurance, and of these about 49% stated that they do not know what the term ‘health insurance’ means, supports this contention. Moreover, the fact that most people live in rural areas while the predominant share of medical facilities resides in urban areas suggests that access to medical care is another significant barrier to achieving good health outcomes [9].

Table 2 provides statistics on self-reported and actual disease rates based on our criteria, for the overall sample and for each state separately. About 43% of the sample had hypertension as measured by the survey staff (denoted by T) but only 17% reported having the condition (S). Of those who were test-diagnosed, 77% reported not having the condition, while of those who tested negative, about 13% reported having it.

**Table 2:** Distribution of test-diagnosed and self-reported disease, by State.

	All States	Punjab	Rajasthan	Kerala	Karnataka
#Households	807	199	191	234	183
1. **BP/Hypertension (0/1)**					
Self-reported (S)	0.17 [0.02]	0.20 [0.05]	0.03 [0.01]	0.33 [0.03]	0.16 [0.04]
Test* (T)	0.43 [0.02]	0.54 [0.02]	0.47 [0.05]	0.33 [0.03]	0.41 [0.03]
False negative (S = 0/T = 1)	0.77 [0.03]	0.75 [0.05]	0.94 [0.02]	0.57 [0.06]	0.74 [0.06]
False positive (S = 1/T = 0)	0.13 [0.01]	0.15 [0.05]	0.01 [0.01]	0.28 [0.03]	0.09 [0.03]
Observations (S)	1,144	285	279	325	255
Observations (T)	1,121	279	266	326	250
2. **Lung disease (0/1)**					
Self-reported (S)	0.04 [0.01]	0.01 [0.01]	0.05 [0.01]	0.09 [0.02]	0.02 [0.01]
Test^@^ (T)	0.43 [0.03]	0.31 [0.05]	0.46 [0.06]	0.43 [0.03]	0.44 [0.05]
False negative (S = 0/T = 1)	0.96 [0.01]	0.99 [0.01]	0.94 [0.03]	0.89 [0.04]	0.95 [0.02]
False positive (S = 1/T = 0)	0.03 [0.01]	0.02 [0.01]	0.04 [0.01]	0.07 [0.03]	0.01 [0.01]
Observations (S)	1,136	284	280	322	250
Observations (T)	577	88	127	206	156

*Note:* Standard errors in parentheses.;

* Systolic > 140 or diastolic > 90 based on average of 3 readings;

^@^ Forced expiratory volume (FEV1) to forced vital capacity (FVC) percentage<70, based on average of 3 readings.

The average under-reporting rate (*S* − *T*) of hypertension in our data, at 26 percentage points, is similar to the estimate of [6] for England, but much higher than the estimates of 3 percentage points for Ontario [7] and 10 percentage points for India [14]. The sample in [14] comprises those aged 18 and above, which could account for the difference since the prevalence of hypertension increases with age [25].

The under-reporting for lung disease is very pronounced in our sample. Only 4% reported having lung disease while 43% of the sample tested positive. Among the latter, 96% reported not having the disease while a negligible share (3%) reported having the disease but tested negative. This finding is at odds with the estimates in [14] and [7], who find that the test-diagnosed rate for lung ailments is marginally less than the self-reported rate. Our sample of valid tests for lung disease is much smaller than that for the self-reports, and this could account for at least some of the differences between our findings and those of other papers. We keep this qualification in mind, and interpret our results for lung function with some caution. Nevertheless, we note that in our sample, the upper bound for false positives is only 4%.

There is notable heterogeneity among states in the actual versus self-reported prevalence of the two conditions. Kerala has a smaller (though still considerable) false negative rate for hypertension at 57 percent, but also a much higher false positive rate than the other states. Rajasthan records an under-reporting rate of 94 percent for hypertension. The under-reporting for lung disease is high across all states. Punjab shows a nearly a 100 percent false negative rate but this is based on a sample of 88 observations only. Overall, false negative reports dominate the reporting errors.

### Measurement error in self-reported health and associated bias

The sample means in columns (1) and (2) in Table 3 are the same as those reported in Table 2. Column (3) is the magnitude of error in *S*. Estimates of the corresponding proportional bias, as reported in column 4, are substantial and similar in magnitude for the two diseases. The bias estimates imply that if we use self-reported hypertension (lung disease) as the control variable instead of the true disease status in any regression analysis, the estimated coefficient will be 83% (87%) smaller than the true coefficient. When we use T1 and T2, the alternate definitions for true hypertension, the associated bias estimates are 91% and 83% respectively, with standard errors of 0.03 in each case. These estimates are very large, and call into question any inference based on using self-reports in place of the true disease status.

**Table 3:** Error decomposition.

Condition	Self-report (S)	Test (T)	Mean error (S-T)	*b*_*us*_
	(1)	(2)	(3)	(4)
1. BP/Hypertension	0.17 [0.016]	0.43 [0.02]	-0.26 [0.024]	0.83 [0.04]
2. Lung disease	0.04 [0.007]	0.43 [0.026]	-0.4 [0.028]	0.87 [0.109]

*Note:* Standard error in parentheses.

Baker et al. report estimates of *b*_*us*_ for 13 health conditions including hypertension, asthma and bronchitis [7]. Their estimates are 0.36 for hypertension, 0.57 for asthma and 0.9 for bronchitis. In comparison, our bias estimate for hypertension is notably bigger, following from the large error in the self-reports. Our *b*_*us*_ estimate for lung disease is very similar to their estimate for bronchitis. Our bias estimate is also significantly bigger than the 0.68 estimate of Johnston et al. for hypertension [6].

Table 4 reports results from OLS and bivariate probit models of reporting errors, to test the hypothesis that self-reports of disease status are unbiased estimators of the true disease status, controlling for individual and household characteristics.

**Table 4:** Tests of hypothesis that error in reporting = 0.

Condition	Hypothesis based on	
	OLS^+^	Bivariate Probit^#^
1. BP/Hypertension	45.11 (0.0)	51.75 (0.0)
2. Lung disease	10.54 (0.0)	1,594.45 (0.0)

^+^ Test statistics are F-tests for the joint significance of the independent variables (and a constant) in a linear regression with dependent variable equal to (S-T), the difference between self-reports (S) and test diagnosis (T) for each condition;

^#^ Test statistics are F-tests for the equality of independent variables in the two probit equations—one using the self-reports (S) and the other using the test diagnosis (T) indicators as dependent variables. The vector of control variables include age, age-squared, female, indicators for state of residence, caste, religion, urban status, marital status, number of children, indicator for whether any child of respondent died, logarithm of household expenditure per capita, literacy status, years of schooling, whether ever worked for pay, indicators for smoking, drinking alcohol and exercise activity, whether respondent is overweight or obese, and whether household has electricity, indoor plumbing, indoor toilet, uses good cooking fuel and test score for memory function.

For both hypertension and lung disease, tests based both on the OLS and bivariate probit specifications unequivocally reject the hypothesis that the self-reports of the disease status are identical to the true disease status in expectation, conditional on the control variables. We therefore estimate models of false negative reporting, to identify characteristics that might be associated with such errors.

### Estimates of false negative reporting

Table 5 presents the marginal effects from censored probit models of false negative reporting for hypertension. We present estimates from two specifications—one controlling for basic socio-demographic characteristics including household income, caste, religion, urban status and state of residence. The second specification also controls for educational attainment, some additional proxies for household income, lifestyle variables, health insurance status and physical risk factors. For comparison purposes, we also present estimates from standard probit models that do not correct for censoring.

**Table 5:** Estimates of false negative reporting: Hypertension.

	*Censored probit*		*Probit*		*Censored probit*		*Probit*	
Variable	Marginal Effect (1)	S.E. (2)	Marginal Effect (3)	S.E. (4)	Marginal Effect (5)	S.E. (6)	Marginal Effect (7)	S.E. (8)
Age	-0.042	0.03	-0.044***	0.012	-0.035	0.022	-0.038***	0.011
Female	-0.017	0.054	-0.027	0.023	-0.03	0.054	-0.057*	0.03
Married	0.011	0.061	0.03	0.029	0.033	0.035	0.035	0.025
Log(HH expenditure pc)	-0.087***	0.03	0.002	0.006	-0.045	0.034	0.005	0.005
Fully literate					-0.097*	0.056	-0.066**	0.025
Schooling (Yrs)					-0.004	0.007	-0.001	0.003
Ever worked					0.012	0.043	-0.014	0.023
Has health insurance					-0.09	0.064	-0.036	0.032
Overweight					-0.057	0.042	-0.072***	0.022
Obese					-0.096	0.073	-0.03	0.039
Waist-to-hip ratio					-0.254	0.159	-0.19**	0.083
Smoking:								
Current smoker					-0.033	0.06	-0.027	0.033
Former smoker					-0.167	0.113	-0.092*	0.053
Drinking:								
Drinks alcohol					0.019	0.056	-0.01	0.036
Formerly drank					0.036	0.078	0.004	0.047
Exercise:								
Exercise often					0.074	0.061	0.027	0.028
Exercise moderately					0.024	0.034	0.026	0.029
HH has electricity					-0.097	0.09	-0.015	0.054
Uses good fuel					-0.107	0.067	-0.083***	0.031
Indoor plumbing					0.137**	0.056	-0.053**	0.021
Toilet inside					0.017	0.068	-0.044	0.035
*ρ*	0.428		-		0.01		-	
	0.391		-		0.649		-	
Wald test for joint significance of instrumental variables (IVs)								
*F*(2, 46)	12.52***		-		12.99***		-	
*Prob* > *F*	0.0		-		0.0		-	
# Observations	1,233		1,265		1,176		1,207	

*Note:* All regressions also control for state of residence, urban status, caste, religion, # children, whether any of respondent’s children died, and memory function score.

***-significant at the 99% level;

**—significant at the 95% level;

*—significant at the 90% level.

For the censored probit estimations, the two instrumental variables (IVs) included in the hypertension equation—pulse rate and an indicator for cardiovascular disease (CVD) risk, denoted as 1 if the C-reactive protein (CRP) concentration in blood is over 3 mg/litre—are statistically significant in both specifications, as measured by the Wald test. However, in both specifications, *ρ*, the estimated correlation between the error terms of the two equations is imprecisely estimated. Moreover, the estimated correlation declines significantly in the extended specification, relative to the basic one. This implies that selection is not a serious concern in our data. In view of this, our preferred specification is the one based on the probit model.

According to the probit estimates in the third column, the propensity to misreport hypertension goes down significantly with age. This is robust to the inclusion of controls for literacy, educational attainment, lifestyle variables and physical risk factors (column 7). Moreover, women have a lower propensity to misreport hypertension. Literacy lowers the propensity to under-report by about 7 percent. However, schooling appears to have no relationship with the dependent variable. Physical risk factors—specifically, being overweight and having a bigger waist-to-hip ratio—also lower the propensity of false negative reporting. This is presumably due to overall poorer health associated with these characteristics, which might require frequent visits to a doctor. The negative effect associated with former smokers is probably a selection effect, reflecting greater awareness and/or self-control—factors that are positively correlated with health in general. Notably, the use of good cooking fuel and availability of indoor plumbing are also associated with a lower probability of under-reporting. These are household characteristics reflecting higher socioeconomic status.

Our finding that women have a lower propensity to misreport is consistent with that of [6]. In contrast to our estimates, however, they find that controlling for censoring in the misreporting of hypertension is important. They also find a small, negative and statistically significant effect of household income on under-reporting. We do not find any evidence of an income gradient.

Table 6 presents the results for lung disease. Since valid spirometry test results are available for a much smaller sample compared to the sample that had blood pressure readings taken, we use a more parsimonious set of control variables than in Table 5. The IVs used in the selection equation—exposure to passive smoking and the level of Epstein-Barr antibody activity in the blood stream—are statistically significant in both specifications at the 5% level, as measured by the Wald test. We again find no evidence of selection in false negative reporting of lung disease. This is even less surprising since there is almost no variation in self-reports among those identified as having lung disease (see Table 2). Our preferred specifications are therefore the probit specifications.

**Table 6:** Estimates of false negative reporting: Lung disease.

	*Censored bivariate probit*		*Probit*		*Censored bivariate probit*		*Probit*	
Variable	Marginal Effect (1)	S.E. (2)	Marginal Effect (3)	S.E. (4)	Marginal Effect (5)	S.E. (6)	Marginal Effect (7)	S.E. (8)
Age	-0.001	0.001	-0.002***	0.001	-0.002	0.001	-0.002***	0.001
Female	0.043**	0.022	0.018	0.013	0.052***	0.018	0.008	0.017
Log(HH expenditure pc)	0.002	0.003	0.003	0.002	0.007**	0.003	0.002	0.002
Fully literate					0.001	0.015	-0.023	0.023
Schooling (Yrs)					-0.001	0.001	0.0	0.002
Overweight					-0.044**	0.017	0.007	0.014
Obese					0.132***	0.039	-0.022	0.023
Ever smoked					0.005	0.01	-0.026**	0.013
Any exercise					0.033**	0.015	0.016	0.013
Uses good cooking fuel					-0.005	0.011	-0.0	0.01
*ρ*	-0.482		-		-0.525		-	
	0.736		-		0.511		-	
Wald test for joint significance of instrumental variables (IVs)								
*F*(2, 46)	4.28**		-		3.55**			
*Prob* > *F*	0.02		-		0.037		-	
# Observations	711		1,257		712		1,255	

*Note:* All regressions also control for state of residence, urban status, caste, religion and memory function score. The expanded specification (Columns 5 and 7), in addition to these variables and those listed, also controls for whether respondent drinks or ever drank alcohol.

***—significant at the 99% level;

**—significant at the 95% level;

*—significant at the 90% level.

As with hypertension, the probit estimations reveal a small, negative and statistically significant effect of age on the dependent variable. There is no evidence of an income gradient or an education gradient. Current and former smokers are about 3% less likely to misreport. This is plausible given the physical symptoms that appear with regular smoking as well as the strong public campaigns to increase awareness of the detrimental effects of smoking. In contrast to the estimates in Table 5, physical risk factors appear not to influence misreporting.

## Conclusions

A large literature documents the relationship between individuals’ health and a number of socioeconomic outcomes. Much of this literature relies on self-reported health as the measure of true health status. More recent work has found big discrepancies between measures of self-reported disease status and corresponding clinically diagnosed measures. This raises concerns regarding the magnitude of bias arising from using self-reported measures in analyses.

We use a cross-sectional wave of health data on older Indians to compare responses for two self-reported health measures, hypertension and lung disease, with their corresponding objective measures. Our analysis makes the assumption that the test diagnosis represents the true health status, and compares the self-reports to this ‘truth’. The results suggest the following: First, self-reported health measures underestimate true disease burden in the population substantially. The estimates of attenuation bias when we use self-reports in regressions are over 80% for each condition. These estimates are much larger than what other studies have found for hypertension. Second, characteristics associated with true disease status have little in common with those associated with self-reports of the same. Third, we find that false negative reporting declines with age. We find no evidence of an income gradient on under-reporting for either disease. There is, however, notable heterogeneity in other characteristics.

We surmise that many people in India are unaware of their true health status due to a mix of factors—lack of knowledge about the nature, causes and symptoms of various diseases, lack of access to medical facilities and a failure of public policies to deliver equitable health services [11, 38, 39]. These factors prevent timely intervention for managing health and controlling disease, invariably leading to morbidity and often to premature death.

We draw two lessons from our results. First, our findings underline the need to supplement subjective health data with comprehensive and reliable clinical measures where possible. Second, there is an urgent need to provide basic health education to citizens, to facilitate access to healthcare and make focused interventions to lower the incidence of disease.

## Supporting information

S1 Table S1 Table gives the definitions of the variables used in the empirical analysis. (PDF)

S2 Table S2 Table gives these statistics for the two alternate definitions of hypertension described in the ‘Materials and methods’ section. When we use the less stringent criterion of more than 160 for systolic or more than 100 for diastolic, the share of those categorized as hypertensive (denoted by T1) falls sharply to 17% in the overall sample. Although the self-reported rate and the test rate are now similar, there is hardly any change in the false negative rates, at 76%. This underlines the extent of the knowledge gap in the sample; changing the thresholds for defining the condition has almost no impact on the false negative report rates. Among the states, only Punjab sees a discernible reduction in the false negative rate based on T1. False positive rates show a small increase relative to the rates based on T, as expected. T2, measured as the average of the second and third blood pressure readings, shows very similar rates of hypertension as the rates based on T, suggesting that the first blood pressure reading is not skewing the test-based diagnosis. The focus of this paper is on false negative reporting, which is similar across the three definitions of hypertension. Therefore, our preferred measure for hypertension is T, based on the globally accepted thresholds of 140/90. However, we test the robustness of our regression-based estimates to the T1 and T2 measures. To check the robustness of our results to alternative definitions of hypertension, we estimated the specifications in Table 5 by using the two different definitions of hypertension, as described in S2 Table. The results were qualitatively similar, and are not reported here.
(PDF)
