# Supplementary material for: The gap between self-reported and objective measures of disease status in India
Source: PLoS One. 2018 Aug 27;13(8):e0202786. doi: 10.1371/journal.pone.0202786 (PMC6110485; doi:10.1371/journal.pone.0202786)
Supplement: S1 Table — (PDF) [file pone.0202786.s001.pdf]

S1 Table Variable Definitions

Variable	Definition
**Basic correlates**	
Age structure	Age categories: 45-54; 55-64; 65-74; 75-85 – categorical variables
Female	=1 if female, 0 if male
Caste	Scheduled castes (SC); scheduled tribes (ST); other backward class (OBC); None/Other – categorical variables
Religion	Hindu; Muslim; Christian; Sikh; Other – categorical variables
Currently married	=1 if currently married, 0 otherwise
# children	Number of living children
Dead_child	=1 if any child of respondent died
Education	Education categories: no education; $<$ 6 years; 6-12 years; $>$ 12 years – categorical variables
Schooling	Years of completed schooling
Literate	=1 if respondent can read and write, 0 otherwise
Ever worked	=1 if respondent is currently working for pay, or worked for pay for at least 6 months in the past, 0 otherwise
**Current circumstances**	
Urban residence	=1 if living in an urban area, 0 otherwise
Log consumption per capita	Log-transformed total household per capita expenditure in last year
Good cooking fuel	=1 if the household uses coal, charcoal, natural gas, LPG, kerosene or electricity for cooking, and 0 otherwise
Good cooking fuel*	=1 if the household uses natural gas, LPG, kerosene or electricity for cooking, and 0 otherwise
Indoor plumbing	=1 if house has indoor plumbing, 0 otherwise
Electricity	=1 if household has electricity, 0 otherwise
Toilet inside house	=1 if household has private toilet, 0 otherwise
**Lifestyle variables**	
Smoking	Never smoked; current smoker; former smoker – categorical variables
Ever smoked	=1 if current or former smoker, 0 otherwise
Drinking	Never drank; current drinker; former drinker – categorical variables
Ever drank	=1 if drinks alcohol or used to, 0 otherwise
Exercise	Never exercised; heavy exercise; moderate exercise – categorical variables
Any exercise	=1 if does heavy or moderate exercise, 0 otherwise
Exposure to passive smoking	=1 if any household member smokes inside the house, 0 otherwise
**Objective health measures**	
Hypertension	=1 if the mean systolic blood pressure measure is over 140 mmHg or the mean diastolic one is over 90 mmHg, and 0 otherwise. Averaged over 3 readings
Lung disease	=1 if ratio of FEV1 (volume of air (in litres) forcibly exhaled in the first second) to FVC (maximum volume of air (in litres) forcibly exhaled out of the lungs until no more can be exhaled) $<$ 70. Based on spirometry test, averaged over 3 readings
Overweight	=1 if the BMI is in the 25-30 kg/m^2 range, 0 otherwise
Obese	=1 if the BMI is $>$ 30 kg/m^2 range, 0 otherwise
Waist-to-hip ratio	Ratio of waist to hip circumference in centimeters
Pulse/heart rate	pulse rate measured using a blood pressure monitor. Averaged over 3 readings
Cardiovascular disease (CVD) risk	=1 if the C-reactive protein (CRP) concentration in blood is $<$ 3 mg/L, 0 otherwise
EBV	Epstein-Barr Virus (EBV) antibody levels
