# Supplementary material for: The gap between self-reported and objective measures of disease status in India
Source: PLoS One. 2018 Aug 27;13(8):e0202786. doi: 10.1371/journal.pone.0202786 (PMC6110485; doi:10.1371/journal.pone.0202786)
Supplement: S2 Table — When we use the less stringent criterion of more than 160 for systolic or more than 100 for diastolic, the share of those categorized as hypertensive (denoted by T1) falls sharply to 17% in the overall sample. Although the self-reported rate and the test rate are now similar, there is hardly any change in the false negative rates, at 76%. This underlines the extent of the knowledge gap in the sample; changing the thresholds for defining the condition has almost no impact on the false negative report rates. Among the states, only Punjab sees a discernible reduction in the false negative rate based on T1. False positive rates show a small increase relative to the rates based on T, as expected. T2, measured as the average of the second and third blood pressure readings, shows very similar rates of hypertension as the rates based on T, suggesting that the first blood pressure reading is not skewing the test-based diagnosis. The focus of this paper is on false negative reporting, which is similar across the three definitions of hypertension. Therefore, our preferred measure for hypertension is T, based on the globally accepted thresholds of 140/90. However, we test the robustness of our regression-based estimates to the T1 and T2 measures. To check the robustness of our results to alternative definitions of hypertension, we estimated the specifications in Table 5 by using the two different definitions of hypertension, as described in S2 Table. The results were qualitatively similar, and are not reported here. (PDF) [file pone.0202786.s002.pdf]

S2 Table Reporting error by state - alternate definitions of hypertension

	All States	Punjab	Rajasthan	Kerala	Karnataka
Self-reported (S)	0.17 [0.02]	0.20 [0.05]	0.03 [0.01]	0.33 [0.03]	0.16 [0.04]
Test[1] (T1)	0.17 [0.02]	0.22 [0.03]	0.19 [0.04]	0.13 [0.02]	0.16 [0.03]
False negative (S=0/T1=1)	0.76 [0.03]	0.69 [0.05]	0.94 [0.03]	0.57 [0.1]	0.71 [0.06]
False positive (S=1/T1=0)	0.15 [0.02]	0.18 [0.05]	0.03 [0.01]	0.31 [0.03]	0.14 [0.04]
Test[2] (T2)	0.41 [0.02]	0.52 [0.03]	0.44 [0.06]	0.34 [0.04]	0.37 [0.02]
False negative (S=0/T2=1)	0.77 [0.02]	0.74 [0.05]	0.93 [0.02]	0.57 [0.05]	0.75 [0.06]
False positive (S=1/T2=0)	0.13 [0.02]	0.14 [0.05]	0.01 [0.01]	0.28 [0.03]	0.11 [0.03]

Note: Standard errors in parentheses.; 1-threshold of 160/100 based on average of 3 readings; 2- threshold of 140/90 based on average of 2nd and 3rd readings.
